# Glycaemic Control for People with Type 2 Diabetes Mellitus in Bangladesh - An urgent need for optimization of management plan

**DOI:** 10.1038/s41598-019-46766-9

**Published:** 2019-07-15

**Authors:** Afsana Afroz, Liaquat Ali, Md. Nazmul Karim, Mohammed J. Alramadan, Khurshid Alam, Dianna J. Magliano, Baki Billah

**Affiliations:** 1 Department of Epidemiology and Preventive Medicine, Monash University, Melbourne, Australia; 2 Bangladesh University of Health Sciences (BUHS), Dhaka, Bangladesh; 3 School of Population and Global Health, The University of Western Australia, Perth, Australia; 4 BakerIDI Heart and Diabetes Institute, Melbourne, Australia

**Keywords:** Epidemiology, Type 2 diabetes

## Abstract

**Aims**: The objective of this study was to identify the determinants of glycaemic control among people with type 2 diabetes mellitus in Bangladesh. A cross-sectional study was carried out during March to September 2017, and 1253 adult patients with type 2 diabetes mellitus were recruited from six hospitals. Data were collected from patients via face-to-face interview, and their medical records were reviewed. Multiple logistic regression analysis was performed. Among the participants, 53.2% were male. Mean (±SD) age was 54.1 (±12.1) years and mean (±SD) duration of diabetes was 9.9 (±7.2) years. About 82% participants had inadequate glycaemic control (HbA1c ≥ 7%) and 54.7% had very poor control (HbA1c ≥ 9%). Low education level, rural residence, unhealthy eating habits, insulin use, infrequent follow up check-ups and history of coronary artery diseases found associated with inadequate and very poor controls. Being female and smokeless tobacco consumer appeared to be associated with inadequate control however cognitive impairment was associated with very poor control only. Prevalence of inadequate glycaemic level was very high in Bangladesh. Having understood relatable lifestyle modification factors, demographics and co-morbidities among people with type 2 diabetes, health care providers in conjunction with patients should work together to address the glycaemic control.

## Introduction

Diabetes is one of the biggest health threats of recent times for the global population, rich and poor alike. It was estimated that, in the year 2017, more than 425 million people globally were suffering from diabetes. The burden is increasing, predominantly in low- and middle-income countries, where about 80% of total diabetes deaths in the world occur^1^. In Bangladesh, the prevalence of diabetes was 9.7% in 2011^2^, and the number of adults with diabetes is projected to be 13.7 million by 2045^1^.

A major concern of diabetes is the complications that accompany it, which occur as detrimental consequences of hyperglycaemia. Long-term hyperglycaemia may cause damage in various organs and can lead to the development of disabling and life-threatening complications such as cardiovascular disease, neuropathy, nephropathy and retinopathy. In 2017, a total of US$727 billion was spent globally to treat and prevent diabetes and its related complications^1^.

The most important aspect of the optimal management of patients suffering from diabetes is prevention of the injurious effects of hyperglycaemia. Glycaemic control is thus considered as the main therapeutic goal for prevention of these severe consequences. Results from many observational studies and randomised controlled clinical trials^3–6^ have revealed that strict control of glycaemic levels helps to prevent complications, especially of the microvascular type, and that these complications are directly related to quality of life^7,8^.

Optimal control of glucose is a daunting task without a clear understanding of the precise dynamics of poor glycaemic control in people with type 2 diabetes (T2DM). Epidemiological studies in different populations^9–14^ have identified several factors that are related to poor glycaemic control. However, most of these studies have been conducted on patients in Western countries. A paucity of such evidence exists for resource-limited populations such as Bangladesh, where diabetes presents a huge burden that goes unnoticed to a large extent. Only one study^15^ has been conducted in Bangladesh attempting to identify the risk factors related to poor glycaemic control; however, it addressed a limited number of factors compared to studies undertaken in Western countries. In order to provide effective research-based evidence, the relationships between poor glycaemic control and all potentially important factors, including compliance with management plans, the effects of lifestyle modification factors, family support, anxiety, depression, and cognitive function, need to be investigated. Thus, the objective of the current study is to estimate the prevalence of glycaemic control, and to widen the investigation regarding related factors, among patients with T2DM in Bangladesh.

## Methodology

### Study design and population

A cross-sectional survey was carried out in Bangladesh from March to September 2017. Data were collected from six hospitals across the country. The selected hospitals are all affiliated with the Diabetic Association of Bangladesh (BADAS), the largest diabetes care provider in the country. All of the hospitals provide various levels of care to people with diabetes. Hospitals affiliated with BADAS are located both inside and outside metropolitan areas across the country, covering rural, urban, and semi-urban settings. Within metropolitan areas, the selected hospitals are the central hospitals that provide primary to tertiary care services. The hospitals outside metropolitan areas usually provide primary to secondary care; hence, patients attending these hospitals but in need of tertiary care are usually referred to the metropolitan hospitals. Using a selection of these specific hospitals for this study allowed for recruiting a heterogeneous sample.

Using 95% confidence level and 5% significance levels, a margin of error of 2.5% for the prevalence of good glycaemic control of 18.8%^15^ obtained from a previous Bangladeshi study on T2DM, a sample size of 938 patients was calculated. However, a total of 1253 patients, aged ≥18 years, all registered with BADAS and having been T2DM patients for a minimum duration of one year, were recruited for the study. The study excluded people suffering from other types of diabetes.

### Data collection

Each patient was interviewed face-to-face using a pre-tested questionnaire after informed written consent was obtained prior to the interview. The questionnaire was pre-tested through a pilot survey in the Bangladesh Institute of Health Sciences hospital. The first section of the questionnaire consisted of questions about patients’ socio-demographic and lifestyle characteristics, including age, gender, marital status, education, profession, monthly household income, smoking status, eating habits, and physical activity. The next section of the questionnaire consisted of clinical and diabetes-related information about the participants such as duration of diabetes, family history of diabetes, frequency of follow-up check-ups, mode of treatment, and hypoglycaemic events. A data extraction checklist was used to collect the data from patients’ records, including laboratory test results, diagnosis, medications, history of complications, and comorbidities.

The final section of the questionnaire was tailored, including the use of several recognised tools to assess a variety of factors known and alleged to relate to aspects of diabetes control. The UK diabetes and diet questionnaire (UKDDQ)^16^ was used, with some minor modifications to make it suitable for the Bangladeshi population. Six selected items from the Global Physical Activity Questionnaire (GPAQ)^17^ were used to assess physical activity levels. Patient Health Questionnaire-2 (PHQ-2)^18^, Generalized Anxiety Disorder Scale (GAD-2)^19^, Michigan Neuropathy Screening Instrument^20^, and a Six-item cognitive impairment test (6CIT)^21^ were used, all without any modification. Permissions were obtained from the respective authorities to use the above-mentioned tools. The weight and height of participants were measured while they were wearing light clothes and no shoes. Waist and hip circumference were measured against thin clothing. Body mass index (BMI) and waist-hip ratio (WHR) were calculated from the collected measurements. Research Electronic Data Capture (REDCap) was used to collect and manage the data^22^.

### Operational definition

The patient’s latest readings (within the previous three months) of glycaemic status were categorised as follows: good glycaemic control = HbA1c < 7%^23^; fair control = HbA1c 7–8%; and poor control = HbA1c > 8.0%^24^. An HbA1c cut-off value of ≥9% was used to represent very poor control^25^. BMI was categorised as: <18.50 = underweight; 18.50–24.99 = normal; >25.00 = overweight or obese^26^. The cut-off point for WHR was defined as a ratio of >0.90 for men and >0.80 for women^27^. Hypertension was defined by either a documented diagnosis of hypertension, the patient taking antihypertension medications, or the latest (within three months) blood pressure readings (either systolic ≥140 or diastolic ≥90)^28^. Dyslipidaemia was defined based on either a documented diagnosis of dyslipidaemia or the patient taking any lipid-lowering medications. Impaired cognitive function was defined as a score of more than seven using 6CIT^21^. Neuropathy was defined as a score of seven or more using the Michigan Neuropathy Screening Instrument^20^. Diabetic foot was confirmed by visual examination of ulcers or amputations and a documented diagnosis of diabetic foot. Physical activity was measured using the GPAQ, and >150 minutes of walking per week was considered as active. Dietary habits were assessed using the UKDDQ^16^. The dietary habit variable was measured on a scale between 0 and 48 and categorised using a cut-off at the first quartile (score 28). A score higher than 28 was considered to indicate healthy eating habits.

### Ethical approval

The project was approved by the Monash University Human Research Ethics Committee and the Ethical Review Committee of the Bangladesh University of Health Sciences. Approval was also obtained from BADAS. All the study procedures were carried out in accordance with the principles of the Declaration of Helsinki as revised in 2013^19^.

### Data management and analysis

Descriptive statistics were reported using mean with standard deviation (SD) for numerical data and relative frequencies (percentages) for categorical data. To test the associations between risk factors and levels of glycaemic control, ANOVA, chi-square tests, and simple logistic regression analysis were used for a univariate analysis. Risk factors with a p-value < 0.05 in simple logistic regression analysis were considered for a multiple logistic regression model. Clinically plausible risk factors were entered into the model even if they appeared to be insignificant in the univariate analysis. Multi-collinearity and the first-order interaction effects between clinically relevant risk factors were investigated. A step-wise backward elimination method was used to select variables that were significantly related to the outcome measures. Multiple logistic regression analysis was mainly performed to identify the determinants for an inadequate glycaemic level (HbA1c ≥7%) as well as for very poor glycaemic control (HbA1c ≥9%). In order to determine the duration-specific risk factors, the patients were stratified by the T2DM durations of: ≤5 years and >5 years^29^. Data were analysed using the statistical software package STATA SE version 15.

## Results

Among the participants, 532 (53.2%) were male. The mean (±SD) age was 54.1 (±12.1) years and the mean (±SD) duration of participants’ diabetes conditions was 9.9 (±7.2) years. The mean (±SD) BMI was 25.7 (±4.9) kg/m² and the mean (±SD) HbA1c was 9.0% (±2.2%). Only 18.8% of participants were classified as having good control, 19.78% had fair control, 62% had poor glycaemic control, and 54.7% had very poor control. Table 1 shows demographic and lifestyle characteristics for various glycaemic levels. The prevalence of poor control was higher among females (p = 0.003), participants with a lower level of education (p = 0.001), those living in rural areas (p < 0.001), and unemployed people or housewives (p = 0.011). Further, the prevalence of poor glycaemic control was higher among smokeless tobacco consumers (p = 0.008) and unhealthy diners (p = 0.008).

**Table 1:** Demographic and lifestyle characteristics by glycaemic control.

Variable	Glycaemic control (HbA1c)			p-value
	Good (<7%) n = 182	Fair (7–7.9%) n = 198	Poor (≥8%) n = 621	
Age in years (mean (±SD)	53.8 ± 12.7	55.9 ± 11.2	53.4 ± 12.2	**0.047**
Age % (n)				
≤40 years	20.5 (31)	13.9 (21)	65.6 (99)	**0.103**
41–60 years	16.7 (93)	19.6 (109)	63.7 (355)	
>60 years	19.8 (58)	23.2 (68)	57.0 (167)	
Gender % (n)				
Female	14.3 (66)	22.1 (102)	63.5 (293)	**0.003**
Male	22.2 (116)	17.4 (91)	60.3 (315)	
Education level % (n)				
Illiterate	14.6 (19)	18.4 (24)	66.9 (87)	**0.001**
Primary	12.1 (21)	18.9 (33)	68.9 (120)	
Secondary	16.5 (73)	20.3 (90)	63.1.(279)	
Tertiary	29.1 (69)	19.4 (46)	51.5 (122)	
Area of residency % (n)				
Rural	14.0 (24)	12.3 (21)	73.7 (126)	**<0.001**
Semi-urban	11.9 (19)	18.8 (30)	69.4 (111)	
Urban	20.8 (139)	21.9 (147)	57.3 (284)	
Working status % (n)				
Employed	233 (7)	26.7 (8)	50.0 (15)	**0.011**
Unemployed	21.9 (79)	15.6 (56)	62.5 (225)	
Homemaker	13.3 (55)	22.3 (92)	64.4 (266)	
Retired	20.9 (41)	21.4 (42)	57.7 (113)	
Income % (n)				
<=20000 tk	14.6 (50)	20.2 (69)	65.3 (223)	0.164
21000–60000 tk	20.1 (85)	17.9 (76)	61.9 (262)	
61000 tk and above	19.9 (47)	22.5 (53)	57.6 (136)	
Active smoking % (n)				
Never	17.2 (129)	21.1 (158)	61.7 (462)	0.336
In the past (>one year)	20.0 (34)	15.9 (27)	64.1 (109)	
Current smoker	23.2 (19)	15.8 (13)	60.9 (50)	
Passive smoking % (n)				
No	18.5 (158)	18.5 (158)	63.0 (537)	0.056
Yes	16.2 (24)	27.0 (40)	56.8 (84)	
Smokeless tobacco % (n)				
Never	20.4 (158)	19.7 (153)	59.9 (465)	0.008
In the past (>one year)	6.4 (4)	19.0 (12)	74.6 (47)	
Current consumer	12.3 (20)	20.4 (33)	67.3 (109)	
Eating habit				
Unhealthy	12.4 (28)	17.3 (93)	70.4 (159)	0.008
Healthy	19.9 (154)	20.5 (159)	59.6 (462)	
Fruits and vegetables % (n)				
Less frequent	17.1 (44)	22.9 (59)	60.1 (155)	0.344
Daily	18.2 (138)	18.7 (139)	62.7 (466)	
Physical Activity % (n)				
Inactive	16.6 (78)	18.5 (87)	64.9 (305)	0.211
Active	19.6 (104)	20.9 (111)	59.5 (316)	

The associations between various clinical characteristics and glycaemic control are presented in Table 2. A higher prevalence of poor glycaemic control was present among people with more than five years’ duration of diabetes (p = 0.005), insulin users (p < 0.001), those having irregular follow-up check-ups (p < 0.001), patients with a history of coronary artery diseases (CAD) (p = 0.001), and those with cognitive impairments (p < 0.001).

**Table 2:** Clinical characteristics by glycaemic control.

Variable	Glycaemic control (HbA1c)			p-value
	Good (<7%) n = 182	Fair (7–7.9%) n = 198	Poor (≥8%) n = 621	
DM Duration years (mean ± SD)	8.1 ± 6.5	10.2 ± 7.5	10.4 ± 7.3	**0.001**
DM Duration % (n)				
≤5 years	24.0 (78)	20.3 (66)	55.7 (181)	**0.005**
6–10 years	17.6 (53)	17.6 (53)	64.8 (195)	
≥11 years	13.6 (51)	21.1 (79)	65.3 (245)	
Family history of DM % (n)				
No	18.4 (120)	19.4 (127)	62.2 (407)	0.921
Yes	17.9 (62)	20.5 (71)	61.7 (214)	
Mode of treatment				
OHA	30.2 (117)	22.4 (87)	47.4 (184)	<0.001
Insulin ± OHA	10.6 (65)	18.1 (111)	71.3 (437)	
Glucometer use % (n)				
Once or more a week	15.5 (38)	21.5 (53)	63.0 (155)	0.508
Less than once a week	18.0 (90)	18.6 (93)	63.3 (316)	
Hypoglycaemia events % (n)				
None	18.9 (149)	19.2 (151)	61.9 (487)	0.724
1–5 times	15.9(30)	21.7 (41)	62.4 (118)	
6 times and more	12.0 (3)	24.0 (6)	64.0 (16)	
Follow up check-up frequency % (n)				
Every 1–3 months	19.5 (144)	21.7 (160)	58.1 (434)	**<0.001**
Every six months	18.3 (17)	23.7 (22)	58.1 (54)	
Annually	12.4 (21)	9.1 (16)	78.2 (133)	
Hypertension % (n)				
No	18.6 (72)	16.0 (62)	65.4 (253)	0.058
Yes	17.9 (110)	22.2 (136)	59.9 (368)	
Dyslipidaemia				
No	17.1 (111)	18.7 (121)	64.2 (416)	0.162
Yes	20.1 (71)	21.8 (77)	58.1 (205)	
CAD % (n)				
No	20.8 (154)	18.3 (136)	60.9 (452)	**0.001**
Yes	10.8 (28)	23.9 (62)	65.3 (169)	
Stroke % (n)				
No	18.2 (169)	20.2 (188)	61.7 (574)	0.479
Yes	18.6 (13)	14.3 (10)	67.1 (47)	
Retinopathy % (n)				
No	10.1 (152)	19.4 (155)	61.5 (491)	0.361
Yes	14.8 (30)	21.2 (43)	64.0 (130)	
Nephropathy % (n)				
No	17.4 (129)	18.9 (140)	63.7 (472)	0.188
Yes	20.4 (53)	22.3 (58)	57.3 (149)	
Neuropathy % (n)				
No	18.5 (174)	19.3 (182)	62.2 (585)	0.301
Yes	13.3 (8)	26.7 (16)	60.0 (36)	
Diabetic foot % (n)				
No	18.4 (167)	19.2 (174)	62.4 (567)	0.304
Yes	16.1 (15)	25.8 (24)	58.1 (54)	
BMI % (n)				
Normal	18.9 (74)	18.2 (71)	62.8 (245)	0.471
Under weight	24.2 (8)	18.2 (6)	57.6 (19)	
Over weight	18.5 (72)	18.2 (71)	63.3 (147)	
Obese	16.9 (21)	26.6 (33)	62.5 (70)	
Family support % (n)				
No	11.5 (7)	13.1 (8)	75.4 (46)	0.085
Yes	18.6 (175)	20.2 (190)	61.2 (575)	
Depression % (n)				
No	19.4 (135)	19.7 (137)	60.9 (424)	0.313
Yes	15.4 (47)	20.0 (61)	64.6 (197)	
Anxiety % (n)				
No	18.6 (167)	19.7 (177)	61.7 (555)	0.630
Yes	14.7 (15)	20.6 (21)	64.7 (66)	
Cognitive function % (n)				
Intact	21.5 (136)	21.4 (133)	57.4 (363)	**<0.001**
Partially impaired	14.7 (11)	22.7 (17)	62.7 (47)	
Impaired	11.9 (35)	16.3 (48)	71.8 (211)	
Waist/hip ratio (mean ± SD)	1.04 ± 0.07	1.10 ± 0.73	1.06 ± 0.08	0.156
Waist circumference (mean ± SD)	95.5 ± 9.1	96.5 ± 9.5	95.8 ± 10.0	0.660

DM: Diabetes mellitus, OHA: Oral hypoglycaemic agent, CAD: Coronary artery disease, BMI: Body mass index.

**Figure 1:**
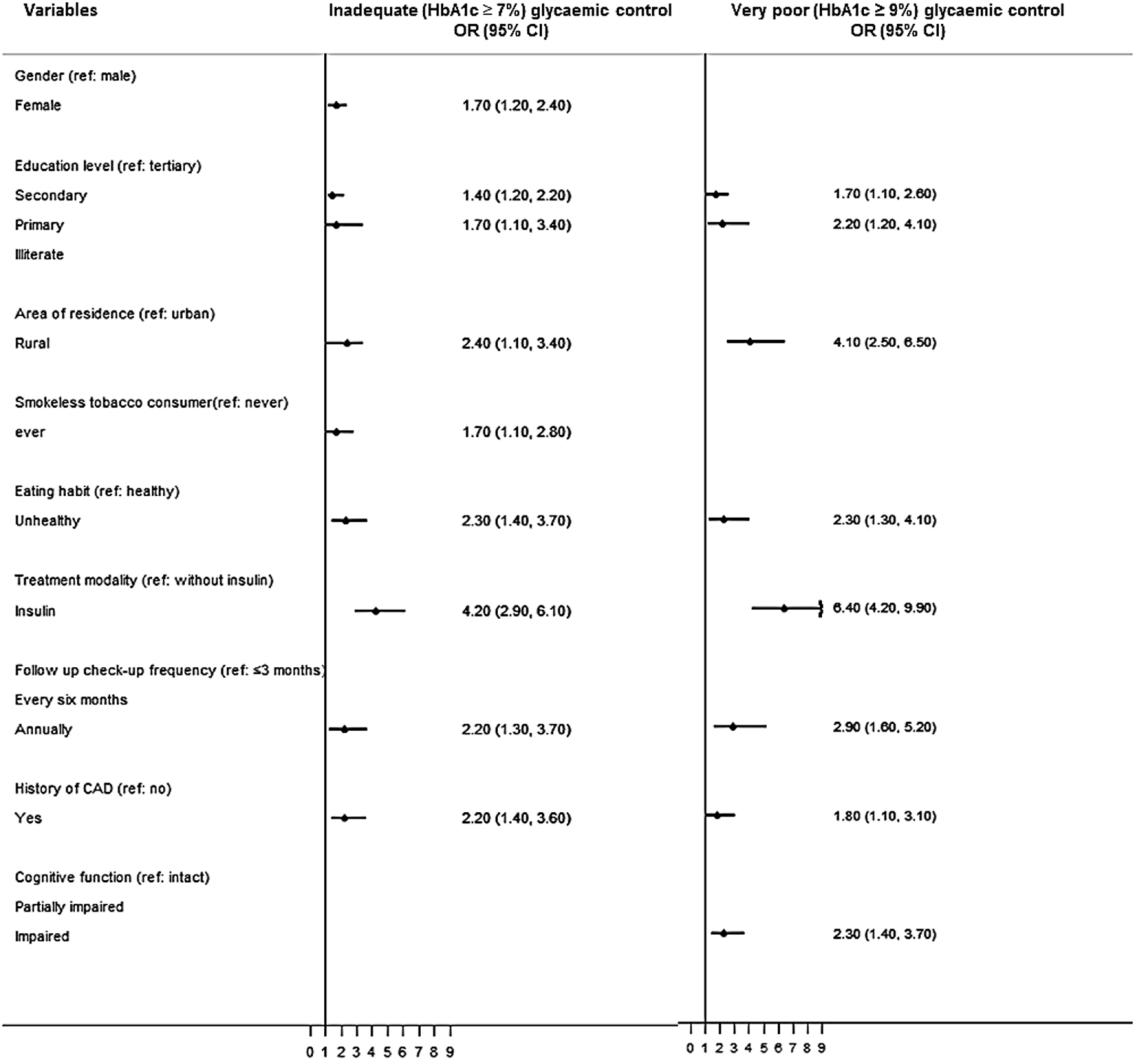
Adjusted association (odds ratio (OR)) between risk factors with inadequate glycaemic control (HbA1c ≥7%) and very poor glycaemic control (HbA1c ≥9%). Note: Variables introduced in to the multiple logistic regression analysis were age, gender, education level, location, work status, income, smokeless tobacco consumption, eating habit, duration of diabetes, modality of treatment, follow up check-up frequency, dyslipidaemia, history of CAD, family support and cognitive function.

The results of multiple logistic regression analysis with step-wise removal for inadequate glycaemic control (HbA1c ≥7%) and very poor glycaemic control (HbA1c ≥9%) are presented in Fig. 1. The following variables presented an increased risk for both inadequate and very poor glycaemic controls: low level of education, residing in a rural area, unhealthy eating habits, use of insulin (either solely or in combination with an oral hypoglycaemic agent [OHA]), infrequent follow-up check-ups, and a history of CAD. However, the majority of these factors had more prominent effects among patients with very poor control. For example, patients living in rural areas showed 2.4-fold (95% CI: 1.1–3.4) higher odds of having inadequate control, compared to 4.1-fold (95% CI: 2.5–6.5) higher odds for very poor control. There were also some differences between risk factors for the two groups: female participants (OR: 1.7, 95% CI: 1.2–2.4) and smokeless tobacco consumers (OR: 1.7, 95% CI: 1.1–2.8) were at higher risk for inadequate control only, while for patients with impaired cognitive functions (OR: 3.2, 95% CI: 1.4–3.7), a higher risk was seen only in the category of very poor control.

The sub-group (duration of T2DM: ≤5 years, shorter and >5 years, longer) analysis results for inadequate and very poor glycaemic controls are presented in Tables 3 and 4 respectively. Table 3 shows that most of the variables in the multiple logistic model appeared as common risk factors between the two groups. However, smokeless tobacco consumption (OR: 2.9, 95% CI: 1.3–6.2) presented a risk only in the shorter duration group and female participants (OR: 2.5; 95% CI: 1.5–4.1) were at higher risk in the longer duration group only.

**Table 3:** Adjusted association (odds ratio (OR)) between risk factors and inadequate (HbA1c ≥ 7%) glycaemic control by duration of diabetes.

Variables	Diabetes duration ≤5 years			Diabetes duration >5 years		
	OR	95% CI	p-value	OR	95% CI	p-value
**Age (ref: ≤40 years)**						
41 – 60 years						
>60 years						
**Gender (ref: male)**						
Female				2.5	1.5-4.1	0.001
**Education level (ref: Tertiary)**						
Secondary						
Primary						
Illiterate						
**Location (ref: urban)**						
Rural	2.5	1.3-4.5	0.002	2.6	1.5-4.7	0.001
**Smokeless tobacco (ref: never)**						
Ever consumer	2.9	1.3-6.2	0.007			
**Eating habit (ref: healthy)**						
Unhealthy	3.1	1.3-6.9	0.007	1.9	1.1-3.3	0.025
**Modality of treatment (ref: without insulin)**						
Insulin	3.8	2.1-7.1	<0.001	4.1	2.6-6.5	<0.001
**Follow up check-up frequency (ref: ≤3 months)**						
Every six months						
Annually	2.6	1.2-5.6	0.016	2.1	1.0-4.3	0.042
**CAD (ref: no)**						
Yes	3.9	1.5-10.3	0.005	1.9	1.1-3.2	0.022
**Cognitive function (ref: intact)**						
Partially impaired						
Impaired						

Note: Variables introduced in to the multiple logistic regression analysis were age, gender, education level, location, work status, income, smokeless tobacco consumption, eating habit, duration of diabetes, modality of treatment, follow up check-up frequency, dyslipidaemia, history of CAD, family support and cognitive function.

**Table 4:** Adjusted association (odds ratio (OR)) between risk factors and very poor (HbA1c ≥9%) glycaemic control by duration of diabetes.

Variables	Diabetes duration ≤5 years			Diabetes duration >5 years		
	OR	95% CI	p-value	OR	95% CI	p-value
**Age (ref: ≤40 years)**						
41–60 years						
>60 years				0.5	0.3-0.8	0.003
**Gender (ref: male)**						
Female						
**Education level (ref: Tertiary)**						
Secondary						
Primary						
Illiterate						
**Location (ref: urban)**						
Rural	3.5	1.7-7.0	**0.001**	4.5	2.3-8.7	0.001
**Smokeless tobacco (ref: never)**						
Ever consumer	3.1	1.2-8.1	**0.010**			
**Eating habit (ref: healthy)**						
Unhealthy	3.1	1.2-8.1	0.017	6.5	3.8-11.2	<0.001
**Modality of treatment (ref: without insulin)**						
Insulin	6.1	2.9-12.6	<0.001			
**Follow up check-up frequency (ref: ≤3 months)**						
Every six months						
Annually	3.3	1.3-7.8	0.007	2.7	1.3-5.9	0.011
**CAD (ref: no)**						
Yes	3.5	1.1-10.8	0.028			
**Cognitive function (ref: intact)**						
Partially impaired						
Impaired				3.2	1.7-6.0	0.001

Note: Variables introduced in to the multiple logistic regression analysis were age, gender, education level, location, work status, income, smokeless tobacco consumption, eating habit, duration of diabetes, modality of treatment, follow up check-up frequency, dyslipidaemia, history of CAD, family support and cognitive function.

The sub-group analysis performed for very poor glycaemic control showed more differentiated risk factors than shared ones between the two groups, as shown in Table 4. Smokeless tobacco consumption (OR: 3.1; 95% CI: 1.2–8.1), use of insulin (solely or in combination with OHA) (OR: 6.1; 95% CI: 2.9–12.6), and history of CAD (OR: 3.5; 95% CI:1.1–10.8) were risk factors in the shorter duration group only, while patients aged above 60 years (OR: 0.5; 95% CI: 0.3–0.8) or suffering from cognitive impairment (OR: 3.2; 95% CI: 1.7–6.0) were at higher risk only in the longer duration group.

## Discussion

Glycaemic control is the cornerstone of managing T2DM and is essential for the prevention of long-term diabetes complications. The key result of this study is that 81.8% of participants did not achieve the recommended HbA1c target of lower than 7%, and 54.7% showed very poor control (HbA1c ≥9%). In general, being female, a low level of education, rural residence, smokeless tobacco consumption, unhealthy eating habits, insulin use, history of CAD, and cognitive impairment were associated with inadequate glycaemic levels in the Bangladeshi T2DM population. Additionally, different sets of risk factors played pivotal roles in the two sub-groups formed based on different durations of T2DM. The cross-sectional nature of the study may not provide reliable insights on temporal relationships; however, the identified association between various factors and glycaemic control is certainly worthy of mention.

The prevalence of inadequate glycaemic control found among people with T2DM is consistent with previous studies conducted in Bangladesh by Selim *et al*.^15^ and Latif *et al*.^30^. The proportion of people with inadequate glycaemic control is higher among females who have had the condition for a longer duration (>5 years), which is also similar to the findings of the previous studies^14,29^. This may be because the females who were in the longer duration group in the current study sample were comparatively older, less compliant with healthy eating, and were being treated with insulin. All of these factors pose individual risks for poorer control. The current study shows that the proportion of people with inadequate glycaemic control is higher among people with a low level of education and those living in rural areas, which is also supported by the previous studies^15,31,32^. People living in a rural area are likely to have a low level of education, and thus have lower levels of T2DM knowledge, low self-management behaviours, low self-efficacy, and low continuity of care. Furthermore, a low level of education increases the likelihood of being in a lower socio-economic class, which may limit their ability to access adequate health care facilities. Due to limited accessibility and affordability, they may not visit a diabetes specialist until they experience complications in the progression of their diabetes. On the other hand, people with a better education and living in urban areas usually have a higher income; thus, they are more likely to be able to afford to receive proper treatment and manage their condition.

The proportion of participants in the current study who are smokeless tobacco users was 22.35%, and the results of the sub-group analysis showed that smokeless tobacco consumers among the shorter duration group were at higher risk of having an uncontrolled glycaemic level. Smokeless tobacco consumption in the form of betel quid (pan) is very common in Bangladesh, especially among women living in villages. Tobacco usually raises blood sugar levels, and the adverse effects of smokeless tobacco on health are evident from previous studies^33^. However, the effect of smokeless tobacco on glycaemic control has not yet been documented in the literature. Thus, an intensive investigation is urgently required in Bangladesh, as well as in other South Asian countries where smokeless tobacco consumption is a common practice. Further, education and awareness programs about the risks of smokeless tobacco consumption could be an option for the management of T2DM.

A healthy lifestyle with regular physical activity and healthy eating habits are very important factors for achieving and maintaining adequate glycaemic control. This study found that participants were active in following the recommended guidelines^28^. Conversely, a relationship was identified between unhealthy eating habits and both inadequate and very poor glycaemic control. Clinical trials^34^ and epidemiological studies^14^ have shown the positive effects of healthy diets on glycaemic control. The majority of the current study’s participants responded that they had received dietary advice from health professionals; however, they also stated that they were unable to maintain the advice. This may be due to a lack of understanding or low affordability of healthy foods. Regular follow-up check-ups add an extra benefit in terms of better control and, similar to previous studies^35^, our findings indicate that regular follow-up check-ups have a positive effect on glycaemic control. Thus, active and individualised education intervention is required to motivate patients towards healthy eating and frequent follow-up check-ups.

Treatment modality also influences the outcome of glycaemic control. In this study, the best glycaemic control was found among participants who used OHA only. Notably, none of the study participants were using insulin pumps or continuous glucose monitoring. A relationship between insulin use and glycaemic control was found in the study results, which is consistent with the findings of other studies^9–11^. Possible reasons for this could include the increasing difficulty of taking multiple medications, injection phobia, or inconvenience of insulin use. Additionally, the efficacy of insulin depends on storage conditions and its proper use, and appropriate dosage in response to different glycaemic levels depends on an individual’s eating habits, exercise regimens, and other lifestyle factors. Insulin users may also have a more progressive disease condition that requires more aggressive treatment, and physicians may attempt dual therapies to achieve better control. Additionally, we found that only 37.6% of our participants used self-monitoring blood glucose devices; this indicates that a lack of proper monitoring, meaning that patients are likely to struggle with managing their insulin. In this context, further research is required to determine why a large proportion of insulin users were identified as having uncontrolled conditions.

Our results also show a relationship between glycaemic control and the presence of CAD. A systematic review investigating factors associated with glycaemic control showed a 48% increased risk of having poor condition control in the presence of CVD^14^. In general, patients with CVD are treated with multiple medications, and some of these may have an adverse effect on glycaemic control. Thus, patients with CVD have a lower likelihood of controlling their condition. The identified relationship between CVD and glycaemic control reinforces the need to optimise the management of CVD.

Another important finding of this study is the association between impaired cognitive function and poor glycaemic control among patients with a longer duration of T2DM. Studies conducted previously in developed countries^36–38^ have shown that there is a strong relationship between poorly controlled diabetes and greater decline in cognitive function. However, no similar study has been conducted in developing countries. Previous literature shows that the toxic effects of chronic hyperglycaemia may be related to slowly progressive functional and structural abnormalities in the brain^39^, and thus could be one of the determinants of cognitive changes in people with diabetes^40,41^; which may not be confirmed by the current study due to its cross-sectional design. Additionally, insulin resistance, at least in the early stages of type 2 diabetes, is associated with compensatory hyperinsulinemia and the consequent accelerated cognitive decline^42^, which supports the findings of our study.

This study has the strength of having a representative population, which increases the likelihood of the generalisability of the findings to people with T2DM attending hospitals associated with BADAS across the country. The study investigated all possible modifiable and non-modifiable factors affecting glycaemic control, which adds strength to the findings. The recruitment of participants and the information collection procedures ensured data quality. However, this study has the limitation of being cross-sectional in design, meaning that a temporal relationship between cause and effect cannot be established.

## Conclusion

In conclusion, the proportion of people with uncontrolled glycaemic levels is considerably high in Bangladesh, which may contribute to an increasing prevalence of complications and thus may pose an extra burden on health care costs. This study has identified a number of modifiable predictors of inadequate glycaemic control: most importantly, different sets of risk factors were identified for participants who had been T2DM patients for different durations. The study’s findings emphasise the importance of being compliant with follow-up check-ups, and of lifestyle modifications, including healthy eating and avoiding smokeless tobacco consumption. A comprehensive knowledge of modifiable and non-modifiable risk factors will help health care providers to individualise the choice of glycaemic goals in reference to duration, with the aim of improving care and outcomes for patients with T2DM.

## Data Availability

The data sets generated during and/or analysed during the current study are available from the corresponding author upon reasonable request.
